# Unpacking the anti-fibrotic arsenal: molecular mechanisms and therapeutic translation of MSC-derived exosomes in pulmonary fibrosis

**DOI:** 10.3389/fimmu.2025.1725041

**Published:** 2025-12-12

**Authors:** Yijia Xiao, Iqra Hoorain, Lin Zhang, Saverio Bellusci, Xuru Jin, Hongzhong Yang, Jin-San Zhang

**Affiliations:** 1 Department of Respiratory and Critical Care Medicine, The Affiliated Changsha Central Hospital, Hengyang Medical School, University of South China, Changsha, China; 2 Medical Research Center, The First Affiliated Hospital of Wenzhou Medical University, Wenzhou, China; 3 Key Laboratory of Interventional Pulmonology of Zhejiang Province, The First Affiliated Hospital of Wenzhou Medical University, Wenzhou, China; 4 Department of Pulmonary and Critical Care Medicine, The Quzhou Affiliated Hospital of Wenzhou Medical University, Quzhou People’s Hospital, Quzhou, China; 5 Institute for Lung Health (ILH), Justus-Liebig University Giessen, Giessen, Germany

**Keywords:** exosomes, mesenchymal stem cells, pulmonary fibrosis, immune modulation, extracellular matrix, drug delivery, targeted therapy

## Abstract

Pulmonary fibrosis (PF) is a progressive, fatal interstitial lung disease with a dire prognosis and limited therapeutic options. Current standard-of-care anti-fibrotic agents (e.g., nintedanib and pirfenidone) offer only modest efficacy in slowing disease progression. Mesenchymal stem cell-derived exosomes (MSC-Exos) have recently emerged as a promising cell-free therapeutic strategy, boasting superior biocompatibility, low immunogenicity, enhanced biodistribution, and an innate tropism for injured tissues. Their potent anti-fibrotic effects are mediated through multiple mechanisms: targeted homing to fibrotic niches; reprogramming of dysregulated immune responses, notably by shifting macrophage polarization from a pro-inflammatory (M1) to an anti-inflammatory/reparative (M2) phenotype; suppression of pathological extracellular matrix deposition via inhibition of core fibrogenic pathways; and alleviation of endoplasmic reticulum stress in alveolar epithelial cells. This review systematically delineates the biological functions and molecular mechanisms underpinning the therapeutic actions of MSC-Exos in PF. We further evaluate completed and ongoing clinical trials (2014–2024), appraise the current translational landscape, and identify persistent challenges in drug development. Ultimately, this integrative analysis aims to define the mechanistic basis of MSC-Exos' efficacy, evaluate their clinical trajectory, and provide a strategic roadmap for their development into precision nanotherapeutics for PF.

## 1 Introduction

Pulmonary fibrosis (PF), a progressive and fatal respiratory disorder characterized by irreversible interstitial scarring, imposes a significant global health burden with increasing morbidity and mortality. Idiopathic pulmonary fibrosis (IPF), the most severe form of PF, has a median survival duration of 2–3 years post-diagnosis, with mortality rates surpassing those of many cancers (1–3). Driven concurrently by population aging and the rising prevalence of established risk factors (e.g., smoking and environmental exposure), the global burden of IPF is projected to increase substantially (4). Despite significant advances in the understanding of the etiology, pathology, and diagnosis of IPF, therapeutic progress has remained limited since the U.S. Food and Drug Administration (FDA) approved pirfenidone and nintedanib over a decade ago (5). While both agents demonstrate efficacy in slowing lung function decline in IPF, critical limitations persist. Neither drug improves patient-reported symptoms and 20–30% of patients exhibit long-term intolerance due to adverse gastrointestinal effects (6, 7). These unmet needs underscore the urgency to develop transformative therapies targeting the multifactorial pathogenesis of the disease.

Exosomes are nanoscale extracellular vesicles (EVs) recognized as pivotal mediators of intercellular communication and immune regulation in addition to playing a role in disease diagnosis and prognosis. Exosome’s ability to directly facilitate the transfer of information and cargo between cells makes them significant carriers of circulating biomarkers (8), and their contents, including nucleic acids, proteins, and other molecular constituents, can profoundly influence the physiological state of recipient cells.

Substantial evidence implicates these vesicles as modulators of the pathogenesis of pulmonary diseases including PF. Exosomes exhibit considerable potential for regulating pulmonary inflammation and attenuating PF progression. They mitigate inflammatory lung injury and decelerate tissue fibrogenesis by conveying anti-inflammatory and anti-fibrotic signaling molecules, thereby suppressing myofibroblast proliferation and activation (9, 10). Additionally, exosomes can readily be obtained from accessible bodily fluids, such as blood, urine, and sputum, making them a promising noninvasive biomarker for PF (11, 12). Mesenchymal stem cell-derived exosomes (MSC-Exos) have demonstrated significant therapeutic efficacy against fibrotic diseases including PF (10, 13, 14). Notably, MSC-Exos possess a distinctive tissue-homing capacity, enabling specific targeting of inflamed or injured sites to promote lung tissue repair (15). Collectively, these findings provide a robust scientific foundation and rationale for developing novel MSC-Exo-based therapeutic agents for PF. This review summarizes the recent advances in understanding the composition, physiological functions, isolation methodologies, and mechanistic roles of MSC-Exos in PF pathogenesis. We further discuss the mechanistic insights into MSC-Exo-based PF therapy, and evaluates the clinical potential and challenges, as well as offering a roadmap for developing next-generation nanotherapeutics.

## 2 Biogenesis and characterization of MSC-Exos

### 2.1 MSC-Exos: biogenesis and isolation paradigms

MSCs originate from the embryonic mesoderm and are a population of multipotent stromal cells characterized by their self-renewal capacity and multilineage differentiation potential. They can be isolated from diverse tissue sources, including the bone marrow, adipose tissue, placental membranes, umbilical cord blood, and dental pulp, with each source conferring distinct biological properties (16). Bone marrow-derived MSCs (BM-MSCs) are particularly valuable because of their robust immunomodulatory and anti-inflammatory properties, whereas adipose tissue-derived MSCs exhibit enhanced differentiation plasticity and migratory capacity, making them advantageous for tissue regeneration applications (17). This source-dependent functional specialization necessitates strategic selection of MSC origins to align with specific therapeutic objectives.

Exosome isolation techniques leverage the unique biophysical and molecular characteristics of these nanovesicles, with methodologies ranging from differential centrifugation and size-exclusion chromatography to immunoaffinity capture, commercial kit-based purification, and microfluidic sorting. Ultracentrifugation remains the gold standard for obtaining high-purity exosome preparations, while emerging commercial kits offer accelerated processing times with comparable yields and purity profiles. Exosomes are characterized using a multimodal analytical approach combining flow cytometry, nanoparticle tracking analysis, transmission electron microscopy, and liquid chromatography-mass spectrometry (LC-MS). Western blotting is further employed to validate specific exosomal markers and cargo composition (18).

**Figure 1:**
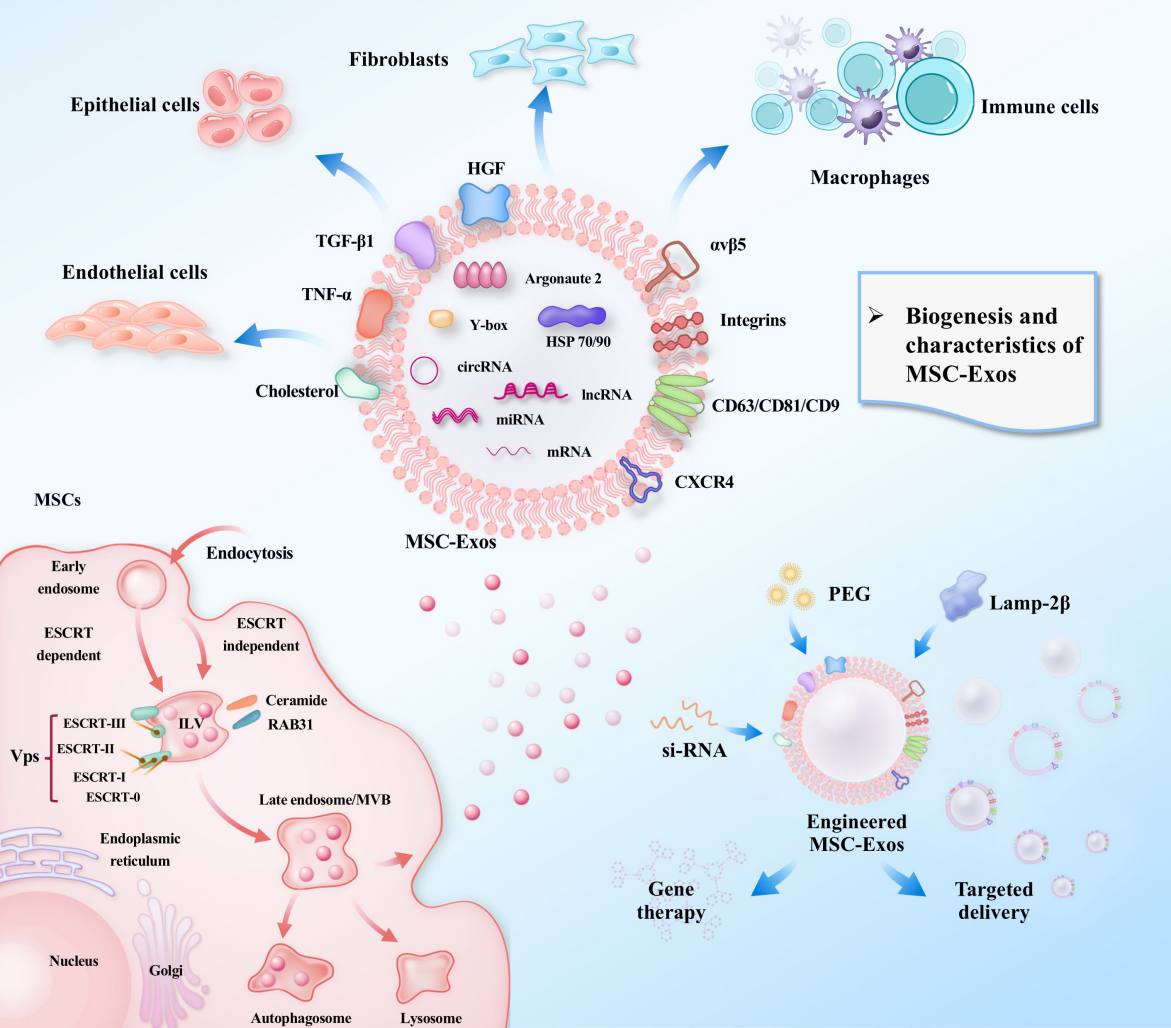
Biogenesis, cargo, and therapeutic targeting of MSC-Exos.This schematic illustrates the biogenesis of MSC-Exos, from intraluminal vesicle formation to secretion. It details their diverse molecular cargo (nucleic acids, proteins, lipids), their interaction with key target cells in the lung (e.g., macrophages, fibroblasts), and strategies for engineering exosomes to enhance their targeted therapeutic potential for treating pulmonary fibrosis.

These nanoscale vesicles (30–150 nm), encapsulated by a lipid bilayer, constitute a dynamic “multimodal signaling hub” containing proteins, nucleic acids (miRNA, lncRNA, mRNA, circRNA), and lipids (**Figure 1**). Proteomic profiling reveals membrane enrichment of tetraspanin family proteins (CD63, CD81, and CD9), homing receptors (CXCR4 and integrin αvβ5), immunomodulatory molecules (HGF, and TGF-β1). The luminal core contains chaperones (HSP70, and HSP90) and RNA-binding proteins (Argonaute 2 and Y-box proteins, AGO2), which stabilize miRNA/mRNA complexes via electrostatic interactions (18).

MSC-Exos exhibit unique functional advantages over tumor- or immune cell-derived exomes. These vesicles are enriched in therapeutic biomolecules, including growth factors, cytokines, and non-coding RNAs, which orchestrate tissue repair while maintaining immune homeostasis. Unlike tumor-derived exosomes, which may promote oncogenic signaling, MSC-Exos demonstrate intrinsic anti-fibrotic and regenerative properties without eliciting pathological immune evasion (19). The evolving understanding of MSC-Exo biology continues to inform therapeutic optimization and standardization strategies for clinical translation.

### 2.2 MSC-Exos contents mediating intercellular communication

The therapeutic potential of MSCs is largely attributable to their robust paracrine activity, with exosomes representing the most extensively studied subclass of EVs. These nanovesicles serve as central carriers that mediate the intricate communication network between MSCs and target cells (**Figure 1**). Enriched in parent cell-derived proteins, lipids, nucleic acids and metabolites, they constitute a sophisticated molecular cargo delivery system. Thus, their roles in communication and therapeutic mechanisms are best understood through examination of their cargo components.

#### 2.2.1 Nucleic acids

MSC-Exos are enriched with diverse cargo nucleic acids, particularly non-coding RNAs such as microRNAs (miRNAs). The selective loading of miRNAs into exosomes is a regulated process mediated by several mechanisms, including AGO2-dependent direct recognition, sphingomyelinase 2(nSMase2)-dependent sorting, microRNA-induced silencing complex (miRISC)-associated pathways, and recognition by SUMOylated heterogeneous nuclear ribonucleoproteins (hnRNPs; primarily hnRNPA2B1, hnRNPA1, and hnRNPC) (20).

The functional impact of exosomal miRNAs is highly context-dependent, varying significantly based on their cellular origin. For instance, miR-125a-5p—one of the most abundant miRNAs in MSC-Exos—is enriched in M2 macrophages and demonstrates cardioprotective effects in heart failure models (21). Similarly, hypoxia-primed MSC-Exos exhibit elevated levels of miR-612, which is associated with pro-angiogenic functions. Conversely, exosomes derived from gastric cancer cells deliver miR-23a to promote tumor angiogenesis (22), while BM-MSC-Exos carry miR-144, which suppresses non-small cell lung cancer proliferation by targeting cyclin E1 and E2.

Exosomal miRNAs also play established roles in fibrotic remodeling. In renal fibrosis models, exosomal miR-21 promotes fibroblast activation via PTEN downregulation (22). Similarly, in cancer microenvironments, tumor-derived exosomal miRNAs (e.g., miR-155, miR-21, and miR-124) induce fibroblast activation and extracellular matrix (ECM) remodeling through targeting factors such as FGF2, TGF-β, and α-SMA (23).

These findings underscore that exosomal miRNA profiles and functions are tightly linked to their cell of origin, ultimately dictating distinct—and often opposing—effects on recipient cells and tissue microenvironments.

#### 2.2.2 Proteins

The MSC-Exos participates in pivotal biological processes, including intercellular communication, cellular structure maintenance, inflammation regulation, and exosome biogenesis. These proteins can modulate disease pathogenesis and facilitate tissue repair and regeneration, paralleling miRNA functionality (24). Anderson et al. (25) identified 1,927 proteins in human BM-MSC-Exos using LC-MS. Ischemic conditions exhibit elevated exosomal levels of angiogenesis-related growth factors, e.g., PDGFs, EGFs, and FGFs, but show constitutive levels of signaling mediators, TNF-α, TGF-β, Wnt5, β-catenin, and delta-like 4. Exosomal protein composition dynamically reflects the parental cell status and adapts to microenvironmental changes. Salomon et al. (26) demonstrated oxygen tension-dependent alterations, where hyperoxic exposure suppressed the exosomal expression of cytoskeletal signaling proteins and the clathrin-mediated endocytosis machinery. This microenvironment-responsive dynamism enables exosomes to actively remodel their extracellular environment.

#### 2.2.3 Lipids

As essential components of exosomes, lipids contribute to membrane formation while critically regulating exosomal biogenesis and release. Exosome formation is governed by two primary mechanisms: endosomal sorting complex required for transport (ESCRT)-dependent and ESCRT-independent pathways (**Figure 1**). The ESCRT machinery, comprising four subcomplexes (ESCRT-0, -I, -II, and-III) assembled from class E vacuolar protein sorting (Vps) proteins, regulates exosomal biogenesis via distinct mechanisms (27). Specifically, phosphatidylinositol 3-phosphate binds to the FYVE domain of ESCRT-0 to recruit early ESCRT proteins (e.g., Vps27/Hrs), thereby promoting exosome formation. The ESCRT-associated protein, ALIX, plays a pivotal role in ESCRT-dependent exosome generation. Studies have demonstrated that ESCRT-III facilitates tetraspanin (CD9, CD63, and CD81) delivery to exosomes via the ESCRT-0–Bro1/ALIX–SNF7/CHMP4 alternative pathway in the presence of lysobisphosphatidic acid, which directly influences exosomal assembly (28). Exosomes also carry lipid-synthesizing enzymes that modulate recipient cell behavior by transferring lipids and lipid-metabolizing enzymes, thereby inducing lipid synthesis in recipient cells while serving as vehicles for the release of cell-synthesized lipids.

In essence, MSC-Exos achieve precise multi-target regulation through their unique molecular compositions and delivery mechanisms. Their tissue-specific cargo encompasses immunomodulatory proteins, selectively packaged non-coding RNAs, and biogenesis-governing lipids, enabling context-dependent signaling. This orchestrated molecular interplay facilitates targeted intercellular communication, positioning MSC-Exos as potent, tunable therapeutic vectors for complex pathologies, such as PF (**Figure 1**).

### 2.3 Exosomes-mediated pathological crosstalk in PF

Intricate intercellular communication is essential for maintaining pulmonary homeostasis and orchestrating responses to injury. This complex network—involving epithelial, mesenchymal, immune, and endothelial cells—becomes profoundly dysregulated in PF, driving a cycle of persistent injury and aberrant repair (29–33). Exosomes emerge as critical mediators of this pathological crosstalk, facilitating the exchange of pathogenic signals that drive disease progression (34, 35). Their role as biomarkers and therapeutic tools underscores their dual utility in both understanding and treating PF.

Through various delicate feedback mechanisms, lung mesenchymal cells reciprocally modulate epithelial function in a subtype-specific manner (36, 37). For instance, Lgr5^+^ mesenchymal cells support AT2 cell expansion via Wnt3a, while Lgr6^+^ cells stimulate the differentiation of airway epithelial progenitors through Wnt-FGF10 signaling. Furthermore, INK4a/P16^+^ mesenchymal cells drive the differentiation of airway progenitors into Club cells via SASP factors (32, 38). This cellular crosstalk becomes dysregulated within the pathological microenvironment of PF, where exosomes emerge as critical mediators of intercellular communication.

Chen et al. (39) demonstrated that in a BLM-induced PF model, exosomes derived from alveolar epithelial cells were enriched in the long non-coding RNA HOTAIRM1, which promotes myofibroblast activation and aberrant ECM accumulation via the miR-30d-3p/HSF1/YY1 axis. Conversely, Secreted frizzled-related protein 1 (Sfrp1), which is highly expressed in myofibroblasts (40, 41) and localizes to active fibrotic regions marked by α-SMA positivity, enhances exosome secretion from fibroblasts. These fibroblast-derived EVs impair alveolar epithelial cell differentiation, thereby exacerbating fibrosis (42). Further highlighting the role of fibroblast-derived EVs, Kadota et al. (43) reported that those carrying miR-23b-3p and miR-494-3p exacerbate mitochondrial DNA damage and senescence in alveolar epithelial cells by inducing mitochondrial dysfunction. In contrast, a protective paracrine mechanism was identified by Xie et al. (44). They found that GHR is highly expressed in mesenchymal cells, with its levels correlating positively with improved lung function in IPF patients. Notably, GHR-rich EVs derived from these cells promoted AT2 cell proliferation and attenuated PF in mice with mesenchymal-specific GHR deficiency. This finding illustrates that paracrine signaling is not exclusive to epithelial cells but is also a critical mechanism used by mesenchymal cells to modulate neighboring cellular responses.

Exosome-mediated intercellular communication significantly influences macrophage function in PF. A key mechanism involves YAP1 activation in fibroblasts, which upregulates CSF1 expression to promote macrophage recruitment and exacerbate fibrotic responses (45). The functional impact of exosomes on macrophages is further illustrated by Feng et al. (46), who demonstrated that AT2 cell-derived exosomes deliver a STIM-activating enhancer to tissue-resident alveolar macrophages. This enhances calcium ion influx and activates the PGC-1α–calcineurin signaling axis, stimulating mitochondrial biogenesis and oxidative phosphorylation. Such metabolic reprogramming alters the macrophage immunophenotype, ultimately attenuating IPF.

The exosomal miRNA cargo is a critical factor in this dialogue (47, 48). Guiot et al. (49) identified a robust increase in exosomal miR-142-3p in sputum and serum from IPF patients, with its levels positively correlating with macrophage abundance. Functionally, miR-142-3p was shown to delay IPF progression by suppressing TGF-β production in both airway epithelial cells and lung fibroblasts, while also reducing the expression of COL1A1 and COL3A1 to mitigate ECM deposition. Beyond epithelial-macrophage crosstalk, inflammatory monocytes regulate mesenchymal cell viability through EVs. This process was found to be mediated by GSDMD activation and pyroptosis (50), revealing another dimension of vesicle-mediated signaling in the fibrotic niche.

Collectively, a propagating cycle of exosome-mediated signaling underpins PF progression. Following epithelial injury, AT2 cells release TGF-β and exosomes that activate myofibroblasts, notably Sfrp1-positive cells. These activated myofibroblasts, in turn, secrete exosomes that further amplify fibrosis and macrophage activation, creating a self-sustaining feedback loop that drives disease exacerbation. This intricate cellular crosstalk is largely coordinated by exosome-derived miRNAs. However, the precise mechanisms by which exosomes orchestrate these intercellular communications following epithelial damage remain complex, tightly regulated, and necessitate further investigation.

## 3 Mechanism underlying MSC-Exos mediated therapy

### 3.1 Immune modulation

**Figure 2:**
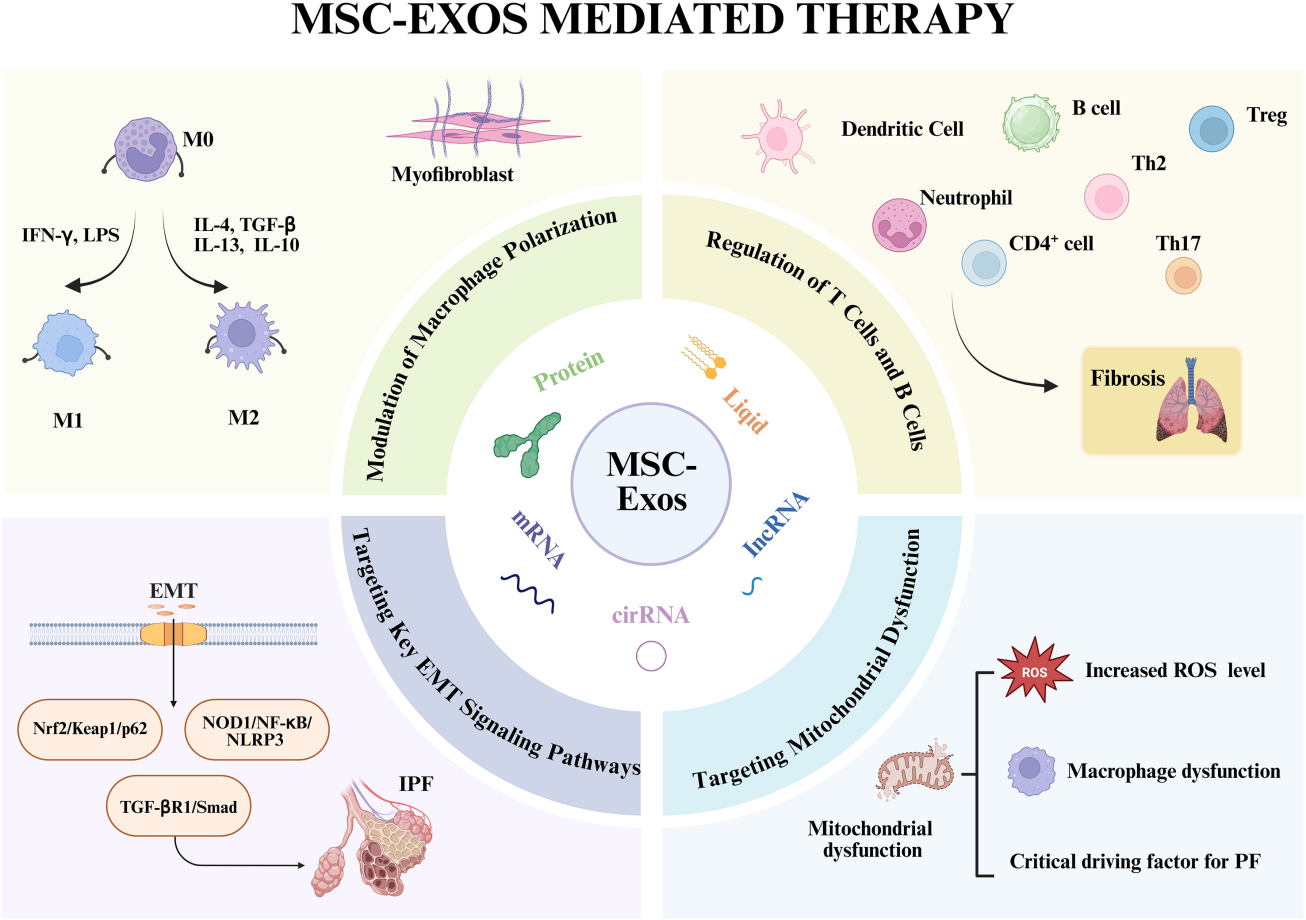
Mechanistic basis for the anti-fibrotic actions of MSC-Exos in PF. The central panel depicts the key molecular constituents of MSC-Exos that mediate their therapeutic effects. The surrounding panels detail the multifaceted mechanisms by which MSC-Exos attenuate PF phenotypes: (i) reprogramming macrophage polarization from a pro-inflammatory (M1) to an anti-inflammatory/reparative (M2) phenotype; (ii) modulating adaptive immune responses by regulating T, B and other cell activity; (iii) inhibiting EMT in alveolar epithelial cells; and (iv) restoring mitochondrial function and reducing ROS level in target cells. Together, these coordinated actions suppress myofibroblast activation and pathological ECM deposition. Created in Biorender. https://BioRender.com

Immune cells are critically involved in the pathogenesis of PF (**Figure 2**) (51, 52). The fibrotic process is initiated when tissue-resident macrophages (TRMs) sense pathogens or damage-associated molecular patterns (DAMPs) via pattern recognition receptors (PRRs), triggering a rapid immune response. This activation prompts the secretion of cytokines and chemokines that recruit inflammatory monocytes, T cells, and fibroblasts, thereby establishing a profibrotic niche (53, 54). Immune modulation is a well-established cornerstone of the therapeutic efficacy of both MSCs and MSC-Exos. Although macrophages are considered primary cellular targets, the precise mechanisms by which MSC-Exos modulate macrophage function to counteract fibrogenesis require further elucidation. Furthermore, emerging evidence underscores the significant role of other immune cell types in mediating the therapeutic effects of MSC-Exos, as detailed below.

#### 3.1.1 Modulation of macrophage polarization

Macrophages represent the most extensively studied and centrally acting immune cells in PF, playing dual roles in both disease initiation and progression (55). During the initial phase of lung injury, classically activated macrophage predominates, secreting substantial quantities of pro-inflammatory cytokines that recruit additional immune cells to the site of damage. When this inflammatory response becomes excessive or fails to resolve appropriately, sustained M1 activation leads to severe tissue damage and epithelial cell death, establishing a foundation for fibrotic initiation. As the disease progresses to more stable stages, alternatively activated M2 macrophages become predominant, directly activating fibroblasts and promoting their differentiation into myofibroblasts through the secretion of pro-fibrotic mediators, including TGF-β and PDGF.

MSC-Exos demonstrate remarkable therapeutic potential by modulating this polarization dynamic, primarily through suppression of early-phase M1 macrophage activation, thereby mitigating inflammatory-mediated tissue damage in pulmonary fibrosis (56). Willis et al. (57) demonstrated that MSC-Exos ameliorate hyperoxia-induced lung injury by regulating pulmonary macrophage polarization, thereby attenuating fibrotic progression, enhancing lung development, and improving vascular remodeling. Dong et al. (58) further elucidated the underlying mechanism, revealing that MSC-Exos achieve these anti-inflammatory and polarization effects by targeting TRAF1, a critical downstream component of the NF-κB/PI3K/AKT signaling pathway. Supporting these findings, Mansouri et al. (59) observed that MSC-Exos treatment increases populations of alveolar macrophages and non-classical monocytes while simultaneously reducing pro-inflammatory monocyte recruitment.

At the functional level, MSC-Exos effectively reprogram myeloid cells toward an immunoregulatory phenotype, reducing the infiltration of pro-fibrotic monocytes into pulmonary tissues. This phenotypic shift translates to decreased collagen deposition, improved lung function, and overall attenuation of fibrotic progression. Molecular analyses reveal that MSC-Exos promote the M1-to-M2 transition through significant downregulation of iNOS coupled with concurrent upregulation of arginase-1 mRNA expression in alveolar macrophages (60, 61). These coordinated changes in key enzymatic markers confirm the polarization shift toward a reparative macrophage phenotype, positioning macrophage polarization targeting as a promising therapeutic strategy for PF intervention.

#### 3.1.2 Regulation of Treg and Th17 cells

Beyond macrophages, MSC-Exos modulate lymphocyte populations central to PF progression (62). They upregulate Foxp3—a master transcription factor for regulatory T cell (Treg) differentiation—in an IDO-dependent manner. This suppresses T helper 17 (Th17) cell differentiation during pulmonary inflammation, thereby inhibiting T-cell-driven lung inflammation and fibrosis (63, 64). The cytokine IL-33 exacerbates PF by promoting myofibroblast activation and inducing the secretion of TGF-β and IL-13 from immune cells such as Th2 and Th17 lymphocytes (65). Xie et al. (66) demonstrated in a bleomycin (BLM)-induced murine PF model that MSC-Exos target IL-33, significantly reducing collagen expression and inhibiting epithelial-mesenchymal transition (EMT).

Tregs (CD4^+^CD25^+^FoxP3^+^ cells) are essential for maintaining immune homeostasis and exert potent anti-fibrotic effects. MSC-Exos can induce Treg production and an anti-inflammatory response by modulating cellular metabolic states (67). Notably, impaired Treg function is a recognized promoter of fibrotic progression in IPF (52). Recent studies have identified a specific MSC subtype characterized by low TNFSF4 expression that effectively induces Treg differentiation in both IPF patients and murine models. These TNFSF4-low MSCs regulate both proliferating (Ki67^+^) and activated (CD38^+^, HLA-DR^+^) Treg populations. The critical role of Tregs was confirmed by antibody-mediated depletion using anti-CD25 treatment, which effectively abolished their anti-fibrotic activity (68).

Although the direct mechanisms of MSC-Exo interactions with Tregs in PF remain to be fully characterized, current evidence indicates that MSC-Exo cargos—including proteins (e.g., IL-6, TSG-6, IDO) and miRNAs (e.g., miR-181c, miR-146a)—promote Treg expansion while simultaneously suppressing inflammatory pathways such as JAK/STAT and TLR/NF-κB signaling.

#### 3.1.3 Effects on dendritic cells

MSC-Exos also modulate the function of dendritic cells (DCs), which are upregulated in the lungs of IPF patients and play a crucial role in antigen presentation (63, 64). Research indicates that MSC-Exos enhance the expression of immunosuppressive IL-10 and TGF-β, inhibit DC maturation, and reduce the expression of co-stimulatory molecules (CD40, CD80, and CD86) on immature DCs. This diminishes the activation of CD4^+^ T cells and Th2-driven fibrotic reactions (63), highlighting how MSC-Exos alleviate inflammatory lung diseases by modulating antigen-presenting cell phenotypes. Together, these immunomodulatory actions foster an immune-tolerant pulmonary microenvironment conducive to fibrosis resolution (69).

### 3.2 Targeting key EMT signaling pathways

EMT is a critical pathological event during the early stages of PF characterized by the loss of epithelial markers (e.g., E-cadherin) and acquisition of a mesenchymal phenotype (e.g., α-SMA, vimentin) in alveolar epithelial cells. MSC-Exos deliver molecular cargo that directly targets and inhibits fundamental EMT signaling pathways driving EMT in these epithelial cells (70). Another study showed that MSC-Exos mitigate BLM-induced PF by modulating the NOD1/NF-κB/NLRP3 pathway, thereby inhibiting EMT (71). These mechanisms are conserved across organs. Cheng et al. (72, 73) established that MSC-Exos attenuate EMT and ameliorate liver fibrosis through YAP signaling-mediated regulation of miR-27b-3p, resulting in downregulation of LOXL2 expression. *In vitro* evidence indicates that MSC-Exos ameliorate liver fibrosis by modulating the Nrf2/Keap1/p62 pathway, leading to the restoration of autophagy and Nrf2 levels, suppression of EMT, reduction of collagen deposition, and attenuation of apoptosis (74). In addition to directly targeting EMT-related pathways, MSC-Exos can also inhibit EMT by modulating cellular functions. Long et al. (75) demonstrated that targeting senescent AT2 cells with high CD38 expression could reverse EMT by restoring NAD+ levels and mitochondrial function, thereby mitigating age-related PF. Similarly, MSC-Exos carrying miR-29b-3p can target FZD6, reduce collagen I and α-SMA expression, and inhibit myofibroblast activity, consequently suppressing EMT formation (76). Notably, exosome-based targeting strategies represent a promising approach for EMT inhibition, which will be discussed in detail in the subsequent sections.

### 3.3 Targeting mitochondrial dysfunction

Mitochondrial dysfunction—characterized by impaired mitophagy, excessive oxidative stress, altered dynamics, and mitochondrial DNA (mtDNA) damage—is a key driver of PF (77, 78). This dysfunction disrupts core cellular functions, including energy production via oxidative phosphorylation (OXPHOS). Mitophagy, a selective form of autophagy for damaged mitochondria, is closely linked to the PINK1/Parkin signaling pathway. While early-stage mitophagy can prevent harmful ROS accumulation, late-stage impairment contributes to fibrotic progression (79, 80). Mitochondrial oxidative stress and mtDNA damage are particularly potent drivers of fibrosis. This dysfunction is closely associated with pulmonary macrophages. Mitochondria isolated from alveolar macrophages of IPF patients exhibit elevated PINK1 and Parkin expression. The protein Akt1 induces mitochondrial ROS (mtROS) production and enhances mitophagy in macrophages; impaired mitophagy in Akt1-knockout and Parkin-knockout mice delays PF progression, suggesting that Akt1 regulates macrophage function via mitophagy to promote fibrosis (81). Furthermore, NADPH oxidase 4 (NOX4) is upregulated in fibrotic lung macrophages, contributing to increased ROS levels that enhance mitophagy and exacerbate PF. PGC-1α is essential for this NOX4-mediated mitochondrial biogenesis (82, 83).

Metabolic reprogramming is fundamental to immune cell behavior. mtROS production is necessary for macrophage polarization, and elevated mtROS activity in the bronchoalveolar lavage of IPF patients correlates with disease severity (84, 85). Profibrotic alveolar macrophages exhibit enhanced glycolysis and tricarboxylic acid (TCA) cycle metabolism, and treatment with glycolysis inhibitors can reverse their phenotype and reduce fibrosis (86). MSC-Exos represent a powerful therapeutic strategy to reverse this mitochondrial dysfunction. Xia et al. (87) demonstrated that AdMSC-Exos can transfer functional mitochondria to alveolar macrophages, enhancing mitochondrial DNA content, membrane potential, OXPHOS activity, and ATP production. This restoration of mitochondrial function promotes a shift toward an anti-inflammatory macrophage phenotype, characterized by reduced pro-inflammatory mediators and increased production of IL-10 and Arg-1, protecting against lung injury. A recent study have demonstrated that FGF21-loaded M2-derived exosomes facilitate M1 to M2 transition by downregulating the expression of glycolysis-related enzymes such as PKM2, PFKFB3, HK2, PDK1, and LDH, and inflammation-related proteins (88). Significantly, MSC-Exos were found to restore alveolar-capillary barrier integrity and normalize oxidative phosphorylation levels via mitochondrial transfer (89).

In summary, MSC-Exos ameliorate fibrosis through multi-faceted mechanisms, including immunomodulation, regulation of EMT signaling pathways, and restoration of mitochondrial function. Importantly, these mechanisms are closely interconnected and strongly associated with immune cells, particularly macrophages. Given the pivotal role of macrophage as targets in mediating the therapeutic actions of MSC-Exos, future research should prioritize investigating the specific interactions between MSC-Exos and macrophages, focusing on: 1/ Molecular Cargo Specificity: Characterizing the key exosomal components (e.g., specific miRNAs, proteins, and lipids) responsible for modulating macrophage polarization and function. 2/ Metabolic Reprogramming: Elucidating the mechanisms and consequences of mitochondrial transfer and other metabolic interventions on macrophage physiology. 3/ Therapeutic Optimization: Engineering exosomes to enhance macrophage-specific targeting (e.g., by displaying specific surface motifs) and exploring synergistic combinations with existing macrophage-centric therapies.

## 4 Exosome engineering strategies for improved therapeutic benefit

Despite their significant therapeutic potential, the clinical application of native MSC-Exos in anti-fibrotic therapy faces considerable challenges (35). A primary limitation is the "off-target effect," largely attributable to rapid phagocytic clearance by mononuclear macrophages of the reticuloendothelial system, leading to unintended accumulation in the liver and spleen (90). To address this limitation and enhance drug bioavailability while minimizing adverse effects, exosome engineering has emerged as a promising strategy to improve targeting precision, drug-loading capacity, stability, and overall therapeutic efficacy.

### 4.1 Surface modification for enhanced targeting

#### 4.1.1 Genetic engineering

A powerful approach involves genetically modifying parent cells to produce exosomes with engineered surfaces primarily aimed at enhancing their targeting specificity. The exosomal membrane, rich in transmembrane proteins such as LAMP-2B, GPI, CD63, CD9, and CD81, can be functionalized by fusing targeting moieties (e.g., peptides, aptamers) to these proteins (91, 92). For instance, the large N-terminal extracellular domain of lysosome-associated membrane protein 2B (LAMP-2B) serves as an excellent fusion scaffold. Long et al. (75) exemplified this by employing chimeric antigen receptor (CAR) technology to generate CD38-targeting EVs from umbilical cord MSCs. Transfection with a lentiviral vector encoding a CD38-specific antigen receptor fused to a CD8 transmembrane domain yielded exosomes that precisely targeted senescent AT2 cells, effectively mitigating age-related pulmonary fibrosis. Furthermore, Zhang et al. (93) have modified MSC-Exos with the SARS-CoV-2 spike protein receptor-binding domain (S-RBD) for treating radiation-induced pulmonary fibrosis. This engineered exosome formulation has been shown to exhibit prolonged retention in lung tissue and significantly improve survival rates while ameliorating pulmonary fibrosis in mouse models.

#### 4.1.2 Peptide-based functionalization

Exosomes can be directly conjugated with tissue-specific peptides to achieve targeted accumulation. The RGD peptide (arginine-glycine-aspartic acid), which exhibits high affinity for integrins (e.g., αVβ3, αVβ5, α5β1) upregulated in fibrotic niches, enables precise delivery to activated fibroblasts and endothelial cells. Further demonstrating this versatility, the octapeptide CC8 (CNGQGEQC) specifically binds integrin α3β1—highly expressed on non-small cell lung cancer cells—making it a suitable ligand for oncology applications (94). Similarly, engineering exosomes with the CRV peptide (CRVLRSGSC), which targets tumor-associated macrophages (TAMs), has been used to create dual-targeting systems capable of homing to cancer cells and TAMs simultaneously, significantly improving anti-tumor efficacy (95). This strategy is readily adaptable to target pro-fibrotic immune cells in the lung.

### 4.2 Delivery of bioactive cargos

Due to their low immunogenicity, inherent biocompatibility, and the lipid bilayer membrane that protects the contents from degradation, engineered exosomes serve as ideal vectors for the targeted delivery of therapeutic molecules.

#### 4.2.1 Delivery of miRNA and siRNA

MSC-Exos naturally carry anti-fibrotic miRNAs, and engineering them to overexpress specific miRNAs offers a potent therapeutic avenue. For instance, Zhang et al. (94) developed MSC-Exos overexpressing miR-486-5p, which combats PF by suppressing Smad2 and activating Akt phosphorylation. BMSC-Exos delivering miR-186 alleviate IPF by inhibiting SOX4 and downregulating DKK1 (96). Exosomes overexpressing miR-29b-3p downregulate Frizzled 6 (FZD6), inhibiting fibroblast activation, differentiation, and proliferation (76). Gu et al. (97) engineered endothelial cell-derived EVs to overexpress miR-125b-5p, enhancing barrier integrity and mitigating acute lung injury. Beyond miRNAs, exosomes can be engineered to deliver siRNA. Cationic lipid (DOTAP)-modified exosomal membranes loaded with Smad4-targeting siRNA (DOTAP/siSmad4@EM) specifically silence Smad4 in lung fibroblasts, exerting potent anti-fibrotic effects (98).

#### 4.2.2 Advanced drug loading and delivery

Utilizing exosomes as drug carriers can enhance drug targeting and drug absorption rate. For example, recognizing the central role of macrophages in silicosis, Chen et al. (99) developed macrophage-derived exosomes loaded with pirfenidone. This system enhanced drug uptake by target cells while reducing systemic adverse effects. To improve drug-cell affinity in PF, fibroblast-derived exosomes co-loaded with bergenin and vitexin significantly reduced collagen deposition and improved lung function in a BLM-induced mouse model (100).

#### 4.2.3 Macrophage-targeted precision therapy

Given the pivotal role of macrophages in orchestrating both the initial inflammatory injury and the subsequent progressive fibrotic cascade (101, 102), they represent a prime therapeutic target for intervention in pulmonary fibrosis. As central regulators of the immune microenvironment, specific macrophage subsets directly drive disease pathogenesis: pro-inflammatory M1 phenotypes mediate early tissue damage and epithelial cell death, while pro-fibrotic M2 and other specialized subsets (e.g., SPP1+) directly activate fibroblasts and promote pathological ECM deposition. The inherent capacity of MSC-Exos to reprogram macrophage polarization validates this cellular target and provides a robust foundation for further precision engineering (59, 101). Future strategies are therefore focused on moving beyond broad modulation to the precision targeting of specific pro-fibrotic macrophage subsets:

##### 4.2.3.1 Targeting M2 macrophages

The macrophage mannose receptor (CD206) is a well-established surface marker for M2-polarized macrophages. Ghebremedhin et al. (103) demonstrated that targeting CD206 significantly attenuates BLM-induced PF. Exosomes engineered with CD206-specific ligands can enable precise delivery of anti-fibrotic cargo to this population.

##### 4.2.3.2 Targeting SPP1^+^ macrophages

SPP1 (osteopontin)+ macrophages represent a distinct, highly pro-fibrotic subset. Engineering exosomes to anchor to SPP1+ macrophages offers another promising strategy for targeted intervention.

##### 4.2.3.3 Targeting metabolic pathways

Metabolites like itaconate are critical regulators of macrophage function in fibrosis. Ogger et al. (104) showed itaconate directly influences human lung fibroblast phenotype *in vitro*, making it and its pathway components attractive targets for exosome-delivered therapeutics.

Collectively, exosome engineering represents a transformative approach overcoming the limitations of native vesicles. By enabling targeted delivery and potent regulation of core fibrotic pathways and the immune microenvironment, engineered exosomes substantially improve treatment precision and efficacy. Future work should focus on optimizing loading efficiency, scaling production, and conducting rigorous preclinical validation. While the methodological approaches are diverse, the overarching goal remains the translation of these advanced therapeutics into clinically effective and precise treatments for pulmonary fibrosis.

## 5 Preclinical and clinical translation

### 5.1 Validity of animal models

The BLM-induced rodent model remains the gold standard preclinical platform for evaluating MSC-Exos in IPF, as it recapitulates key pathological features of human disease, including fibroblast-to-myofibroblast transition, sustained alveolar inflammation, and progressive ECM remodeling. However, this model cannot fully capture the complexity and chronicity of human IPF. Notably, the therapeutic potential of MSC-Exos extends beyond the BLM model, demonstrating robust anti-fibrotic efficacy in silica-induced silicosis, radiation-induced pulmonary fibrosis, and other cross-species models (74, 87, 89, 105). This pan-fibrotic therapeutic effect, coupled with an excellent biosafety profile, positions MSC-Exos as a transformative therapeutic strategy for a spectrum of organ-specific fibrotic diseases that share common pathogenic pathways. These compelling preclinical data provide a critical scientific foundation for clinical translation.

### 5.2 Progress in clinical trials

Although clinical trials investigating MSC-Exos for pulmonary diseases are still in early stages, they have demonstrated promising therapeutic potential. To date, most MSC-Exo-based clinical trials for pulmonary conditions focus on Acute Respiratory Distress Syndrome (ARDS) and COVID-19-related lung injury, where they have shown notable efficacy (**Table 1**). Several pioneering clinical trials using MSC-derived therapies for IPF have also been conducted, which have yielded valuable safety data and paved the way for future EV-based applications (**Table 2**).

**Table 1:** Summary of ongoing and completed MSC-EV clinical trials in pulmonary diseases.

Trial identifier	MSC-type	Condition	Trial phase	Study type	Status	Route	Dose	Ref.
NCT04544215	MSC-Exos	Pulmonary infection	Phase I & II	Interventional	Completed	AI	Low: 8.0×10^8^ exosomes/3 ml; High: 16.0×10^8^ exosomes/3 ml	(113)
NCT04276987	MSC-EVs / haMSC-Exos	COVID−19 related Pneumonia	Phase I	Interventional	Completed	AI	5 times of MSC-Exos (2 × 10^8^ nanovesicles/3 ml at 1–5 days	(120)
NCT04313647	haMSC-EVs in volunteers	Lung injury	Phase I	Interventional	Completed	AI	2 × 10^8^ to 16 × 10^8^ particles	(121)
NCT04602104	hMSC-Exos	ARDS	Phase I & II	Interventional	Completed	AI	Low: 2.0 × 10^8^ exosomes; Medium: 8.0 × 10^8^ vesicles; High: 16.0 × 10^8^ exosomes	(122)
NCT05216562	MSC-Exos	COVID‐19	Phase II & III	Interventional	Completed	IV	Standard	(123)
NCT03857841	BM−MSC EVs	BPD	Phase I	Interventional	Completed	IV	20/40/60 per mol phospholipid/ Kg	(124)
NCT04493242	BM−MSC EVs	COVID-19 ARDS	Phase II	Interventional	Completed	IV	Two doses of 15 mL	(125)
IRCT20200217046526N2	MSC-Exos	COVID-19 ARDS	Phase II	Interventional	Completed	IV	One dose of MSCs (100 × 10^6^ cells) followed by one dose of MSC-EVs	(126)
ChiCTR2000030261	MSC-Exos	COVID-19 pneumonia	Phase I	Interventional	Completed	Nb	Different doses to 8 patients	(127)
ChiCTR2300075466	hUCMSC-EVs	COVID-19 pneumonia	Phase I	Interventional	Completed	Nb	2 × 10^9^ particles per person	(109)

ARDS, acute respiratory distress syndrome; BPD, broncho-pulmonary dysplasia; BM-MSC-Exos, bone marrow-derived MSC-Exos; haMSC-Exos, human adipose-derived MSC-Exos; hUCMSCEVs, human umbilical cord-derived MSC-derived EVs; AI: aerosol inhalation; IV, intravenous; Nb, nebulization.

**Table 2:** List of selected clinical trials on the use of MSC-EVs in pulmonary disease treatment.

Trial identifier	MSC-type	Condition	Trial phase	Study type	Status	Route	Dose
NCT04602442	MSC-Exos	SARS-CoV-2 Pneumonia	Phase II	Interventional	Unknown	AI	0.5-2×10^10^ exosome/3 ml, twice a day for 10 days
NCT05191381	MSC-EVs	Fibrosis after COVID-19	–	Observational	Ongoing	IV	Application of exosomes in a whole blood assay
NCT04491240	MSC-Exos	SARS-CoV-2 pneumonia	Phase I & II	Interventional	Completed	AI	Twice a day, contained 0.5–2 × 10^10^ of nanoparticles
NCT05354141	BMMSC-EVs	ARDS	Phase III	Interventional	Completed	IV	Multicenter, randomized, double-blinded, placebo-controlled trial
NCT05787288	MSC-EVs	COVID‐19 pneumonia	Phase I	Interventional	Completed	AI	1 × 10^9^ particles/ml
NCT05127122	BMMSC-EVs	ARDS	Phase I & II	Interventional	Completed	IV	10 mL or 15 mL of ExoFlo
NCT05125562	BMMSC-EVs	COVID‐19	Phase II	Interventional	Completed	IV	7 × 10^11^ to 10.5 × 10^11^ of particles once
NCT04798716	MSC-Exos	ARDS	Phase I & II	Interventional	Completed	IV	1^st^ Dose: MSC-Exos (2:4:8); 2^nd^ Dose: MSC-Exos (8:4:8); 3^rd^ Dose: MSC-Exos (8:8:8)
NCT05808400	BMMSC-EVs	COVID‐19	Phase I	Interventional	Completed	AI	1x 10^9^ particles/ml.
NCT05116761	BMMSC-EVs	COVID‐19 syndrome	Phase III	Interventional	Completed	IV	15 mL of ExoFlo, approximately 10.5 × 10^8^ of particles (mixed with 85 mL of normal saline)

Data accessible at https://clinicaltrials.gov/ct2/home

#### 5.2.1 NCT05191381

Up to the present, one registered clinical trial has been reported investigating MSC-EVs therapy for pulmonary fibrosis. The ongoing study initiated in 2021 includes participants aged 18–90 years for intravenous administration of MSC-EVs with pulmonary fibrosis after COVID-19 (106).

#### 5.2.2 NCT01385644

This phase 1b, open-label, dose-escalation trial evaluated the safety of intravenous administration of placenta-derived MSCs in 8 patients with moderate to severe IPF. The intervention was found to be safe and well-tolerated at doses up to 2 × 10^6^; cells/kg, with the majority of adverse events being mild and self-limiting. No significant changes in lung function, 6-minute walk distance, or CT fibrosis scores were observed over the 6-month follow-up (107).

#### 5.2.3 ETHER (NCT02013700)

This single-center, non-randomized phase I study assessed the safety of a single intravenous infusion of allogeneic bone marrow-derived MSCs (20, 100, or 200 × 10^6^; cells) in nine patients with mild-to-moderate IPF. The primary endpoint was the incidence of treatment-emergent serious adverse events (SAEs) within 4 weeks. No SAEs were reported, reinforcing the short-term safety of allogeneic MSC infusion and building confidence for future cell therapy trials in lung diseases (108).

#### 5.2.4 The MR-46-22-004531 (ChiCTR2300075466)

This recent randomized, single-blind, placebo-controlled phase 1 trial involved 24 patients with PF of heterogeneous etiologies. Based on robust preclinical data in a BLM mouse model that identified miRNA-486-5p-mediated macrophage polarization as a key mechanism, the trial administered nebulized hUCMSC-EVs (2×10^9^ particles/person). The treatment was well-tolerated with no severe adverse events. The experimental group showed significant improvements in pulmonary function (FEV1, FVC, MVV, DLCO), respiratory scores, and quality of life. Notably, marked radiological regression was observed in two patients with advanced post-inflammatory PF, while a control patient exhibited progression, suggesting a potential etiology-specific therapeutic benefit (109).

## 6 Clinical challenges and translation to therapy

Despite their significant promise as a next-generation therapeutic platform, several formidable challenges must be systematically addressed to facilitate the successful clinical translation of MSC-Exos for PF Therapy.

First, a critical challenge is the lack of standardized, scalable, and reproducible protocols for the isolation, purification, characterization, storage, and transport of MSC-Exos. Established isolation methods include ultracentrifugation, ultrafiltration, immunoaffinity capture, polymer-based precipitation via charge neutralization, size-exclusion chromatography, and microfluidic techniques. Each approach presents distinct advantages and limitations, as comprehensively reviewed by Nobendu Mukerjee et al. (110). Ultracentrifugation, the “gold standard,” yields high-purity exosomes but with lower overall output, whereas polymer-based precipitation provides higher yields at the cost of purity, reflected by its predominant use in published clinical trials. MSC-Exos characterization must adhere to the minimal experimental requirements for extracellular vesicles outlined in the MISEV2023 guidelines, encompassing specific marker expression and physical properties. Nearly all MSC-Exos express typical markers such as CD9, CD63, CD81, and TSG101, while lacking Calnexin and Cytochrome C (111). Techniques like electron microscopy and nanoparticle tracking analysis are most widely employed. Scaling from small preclinical batches to clinical-grade quantities requires advanced bioreactor systems while maintaining exosome integrity and bioactivity (112). Aggregation during production or storage can compromise therapeutic efficacy and pharmacokinetics. Variability in cell sources, culture conditions, purification, and isolation, affects the particle size, purity, and cargo composition to predominantly enhance the progression of clinical trials (110, 113). Hence, rigorous quality control metrics and standardized operating procedures (SOPs), despite European Medicines Agency (EMA), U.S. Food and Drug Administration (FDA) guideline for EVs is limited (114, 115). Furthermore, scaling up from laboratory research to GMP-compliant industrial manufacturing—while maintaining consistent therapeutic potency and purity—remains a pivotal obstacle in clinical translation and eventual commercialization (116).

Second, a comprehensive delineation of the complete pharmacological and safety profile of MSC-Exos is imperative. Critical aspects such as their potential toxicity, immunogenicity, biodistribution, clearance mechanisms, and long-term biosafety—particularly following repeated administration to diseased lung tissue—remain inadequately characterized. Essential pharmacological parameters, including the optimal therapeutic dosage, treatment frequency, pharmacokinetics, pharmacodynamics, and the most effective route of administration, must be rigorously established through systematic and well-designed preclinical and clinical studies (117, 118).

MSC-Exos are quantified by employing different metrics, some rely on protein concentration measurements (93, 119). However, that the highest tolerable dose does not necessarily equate to therapeutic efficacy. BCA assay method offer rapid and convenient protein quantification, directly reflecting the total protein content but is susceptible to inaccuracies. In contrast, particle number quantification, a more direct assessment of vesicle count, evaluate exosome yield and standardizing dosing regimens. Another crucial factor is the route of administration influencing the efficacy of MSC-Exos. Intravenous injection is the most commonly used method in preclinical studies for pulmonary diseases (113). For instance, nebulized inhalation represents the preferred route for targeted lung delivery, as it enables direct deposition of the therapeutic agent to the pulmonary region.

Furthermore, MSC-Exos therapeutic effects appear to involve a complex, multi-factorial "cocktail effect" resulting from the synergistic action of myriad molecular components (e.g., miRNAs, proteins, lipids). Key mechanistic questions remain unanswered: Are certain specific miRNAs (e.g., miR-21-5p, miR-let-7 family) playing dominant roles? How do specific cytokines or lipids contribute to the overall anti-fibrotic and immunomodulatory effect? The precise interaction networks between exosomes and various recipient cell types (e.g., fibroblasts, myofibroblasts, macrophages, epithelial cells) within the complex and dynamic PF microenvironment are still poorly characterized. Additionally, PF (including IPF and secondary PF) exhibits considerable patient-to-patient heterogeneity. Individuals with different disease etiologies, genetic backgrounds, and clinical stages likely have distinct molecular driving mechanisms, yet we have not identified which specific patient subsets are most responsive to MSC-Exos therapy. Predictive biomarkers for stratifying patients and forecasting treatment efficacy are currently lacking, hindering the development of personalized treatment approaches.

The path to clinical translation is undoubtedly complex; however, the encouraging safety and efficacy outcomes from early-phase clinical trials affirm the considerable clinical potential of MSC-Exos (109). A concerted effort to overcome the hurdles of production standardization, scalable manufacturing, rigorous quality control, and comprehensive safety assessment will be absolutely essential for the successful development and widespread clinical application.

## 7 Conclusion and perspective

In summary, MSC-Exos represent a transformative therapeutic paradigm for PF, capitalizing on their multifaceted mechanisms of action and favorable biosafety profile to address fundamental limitations of conventional treatments. Current research underscores that the pathophysiological status of parent cells critically determines exosomal cargo composition, emphasizing the necessity for stringent quality control protocols and standardized characterization of source materials. Despite all preclinical evidence in animal models, clinical translation of MSC-Exo therapy for PF remains limited. So far, no peer-reviewed publications mentioning completed clinical studies in this specific therapeutic domain have emerged.

Future investigations should prioritize several pivotal research directions (1): development of advanced biocompatible engineering strategies to enhance tissue-specific targeting and precision (2); implementing integrated multi-omics approaches to delineate mechanistic pathways and identify validated biomarkers for patient stratification (3); optimizing versatile loading methodologies to facilitate synergistic combination therapies with diverse therapeutic modalities (4). viable alternatives for pretreatment strategies to exhibit excellent biocompatibility and significantly potentiate regenerative outcomes. Addressing these challenges through rigorous mechanistic studies, harmonized quality-control frameworks, and well-designed clinical trials will be essential.

The clinical translation pathway for MSC-Exos is strengthened by their exceptional stability, minimal immunogenicity, modular and tunable delivery capabilities, effectively circumventing risks inherent to whole-cell therapies. As mechanistic insights deepen and manufacturing technologies advance, these exosome-based therapeutics are poised to transition from investigational agents to foundational elements of precision medicine for fibrotic lung disorders, potentially addressing critical unmet needs in treatment-refractory cases.
